# A Comparative Evaluation of the Chemiluminescence Immunoassay and ELISA for the Detection of Islet Autoantibodies in Type 1 Diabetes

**DOI:** 10.3390/diagnostics15131695

**Published:** 2025-07-03

**Authors:** Elisa Danese, Claudia Piona, Mariateresa Rizza, Elena Tiziani, Laura Pighi, Elisa Morotti, Gian Luca Salvagno, Camilla Mattiuzzi, Claudio Maffeis, Giuseppe Lippi

**Affiliations:** 1 Clinical Biochemistry Section, Department of Engineering for Innovation Medicine, University of Verona, Strada le Grazie 15, 37134 Verona, Italy; elisa.danese@univr.it (E.D.); mariateresa.rizza@studenti.univr.it (M.R.); elena.tiziani@studenti.univr.it (E.T.); laura.pighi2@aovr.veneto.it (L.P.); gianluca.salvagno@univr.it (G.L.S.); 2 Section of Pediatric Diabetes and Metabolism, Department of Surgery, Dentistry, Pediatrics, and Gynecology, University of Verona, Piazzale L.A Scuro 10, 37126 Verona, Italy; claudia.piona@univr.it (C.P.); elisa.morotti@univr.it (E.M.); claudio.maffeis@univr.it (C.M.); 3 Medical Direction, Rovereto Hospital, Provincial Agency for Social and Sanitary Services (APSS), Corso Verona, 4, 38068 Rovereto, Italy; camilla.mattiuzzi@apss.tn.it

**Keywords:** chemiluminescence immunoassay, CLIA, ELISA, GADA, ZnT8A, IA-2A, islet autoantibody, type 1 diabetes mellitus

## Abstract

**Background:** The early detection of type 1 diabetes (T1D) through screening for major islet autoantibodies is receiving increasing attention as a public health strategy, exemplified by the recent implementation of a pilot pediatric screening program in Italy. The transition from research-based screening to large-scale population initiatives needs automated and standardized assays that are capable of processing extensive sample volumes. Hence, this study aimed to evaluate the analytical performance and comparability of a fully automated chemiluminescence immunoassay (CLIA) compared to a conventional enzyme-linked immunosorbent assay (ELISA) for the detection of three classes of major islet antibodies—anti-GAD (GADA), anti-IA-2 (IA-2A), and anti-ZnT8 (ZnT8A). **Methods:** A total of 104 serum specimens were analyzed for each autoantibody using both ELISA (RSR and Medyzim, DYNES, DSX) and CLIA (MAGLUMI 800). Assay precision and linearity were assessed through intra-assay variability studies and dilution protocols. Methods agreement was evaluated with Passing–Bablok regression, Spearman’s correlation, Bland–Altman analysis, and Cohen’s kappa statistics. **Results:** The CLIA showed good precision and excellent linearity across clinically relevant concentration ranges of all islet antibodies. Correlation coefficients and categorical agreement between CLIA and ELISA were high (r > 0.96 and Cohen’s kappa >0.8 for all), with ZnT8A exhibiting the highest concordance. However, proportional biases were found, as CLIA systematically underestimated GADA and ZnT8A levels, while overestimated IA-2A compared to the ELISA. **Conclusions:** The CLIA displayed satisfactory precision and agreement with ELISA for GADA, IA-2A, and ZnT8A detection. Our findings support the use of these automated immunoassays in large-scale population initiatives for diagnosing T1D, but we also highlight the need for further efforts to achieve better inter-assay harmonization.

## 1. Introduction

Screening for type 1 diabetes (T1D) is gaining international recognition as a potential component of standard preventive care. While current efforts are largely research-based, several countries are initiating structured implementation strategies. In 2023, Italy became the first country to formally enact a public health policy mandating population-wide pediatric screening for both T1D and celiac disease (CD). The Istituto Superiore di Sanità (ISS., i.e., the National Health Institute), under the directive of the Ministry of Health, launched a government-funded pilot program—D1Ce (Diabete tipo 1 e Celiachia) Screen—across four regions (Lombardia, Marche, Campania, and Sardegna) [1]. This initiative aims to assess the feasibility, acceptability, and operational challenges of large-scale screening, with a major focus on sample collection and autoantibody detection, serving as a foundational step toward a future nationwide implementation [1].

The main purpose of T1D screening programs is to detect individuals at risk or in early disease stages, enabling preventive interventions. The demonstrated benefits include a significant reduction in diabetic ketoacidosis (DKA) at diagnosis (i.e., from 15–80% to below 5%), with a consequent improvement in acute morbidity, neurocognitive impairment, and mortality [2]. Screening also improves short-term clinical outcomes and reduces hospitalization, contributing to better psychological adjustment, as parents of screened children report lower anxiety levels at diagnosis [3]. Beyond these immediate benefits, recent advances in immunotherapy, such as the approval of teplizumab for delaying the onset of clinical T1D, have expanded the relevance of early identification strategies. These developments have renewed interest in the feasibility and potential impact of population-based screening programs, aiming not only to improve diagnosis, but also to enable timely preventive interventions [4,5].

The assessment of islet autoantibodies, recognizing four major pancreatic autoantigens, is now clinically available, comprising anti-insulin autoantibodies (IAAs), anti-glutamate decarboxylase autoantibodies (GADAs), anti-insulinoma antigen-2 autoantibodies (IA-2As), and anti-zinc transporter protein 8 antibodies (ZnT8As) [6,7]. These tests represent the screening targets recommended by the most recent American Diabetes Association (ADA) Standards of Care [8]. The presence of two or more autoantibodies in the context of normoglycemia defines Stage 1 T1D. Stage 2 is instead characterized by the same serological profile in association with dysglycemia, in the absence of clinical symptoms. Stage 3 corresponds to the clinical onset of T1D, with hyperglycemia meeting diagnostic criteria established by the ADA [9,10].

Various methods have been proposed for the detection of anti-TDM1 antibodies, including the radioimmunoassay (RIA) and the enzyme-linked immunosorbent assay (ELISA) [11]. Although the RIA technique is extremely sensitive and specific, it requires specialized equipment, special precautions, and licensing since radioactive substances are used. For this reason, most laboratories worldwide are increasingly abandoning this analytical technology and the ELISA has emerged as the primary detection technique due to its quantitative capabilities and straightforward procedure [12,13]. Nonetheless, the ELISA still possesses certain limitations inherent to traditional immunological detection methods, such as the need for multiple operational steps, extended time requirements, and high costs [14]. Additionally, achieving rapid sample detection in clinical settings can be challenging with the ELISA [15]. To address these limitations, chemiluminescence immunoassay (CLIA) detection technology has gained popularity as a new and mainstream clinical immunoassay method [16]. The CLIA comprises the following steps: (a) labeling an antigen or an antibody with a chemiluminescence-related substance; (b) separating a free chemiluminescence-related marker after a specific antigen–antibody reaction; (c) adding other related substances of a chemiluminescence-related system to generate chemiluminescence; and (d) performing qualitative or quantitative detection on labeled antigens or antibodies. The CLIA has gained widespread use in clinical disease diagnosis, particularly for detection of cardiac or tumor biomarkers and autoantibodies, owing to its rapid detection, ease of operation, and high sensitivity and specificity. As a result, it could be applied in pharmaceutical control, clinical diagnostics, and environmental monitoring. It is now regarded as the best alternative to the ELISA and RIA [17].

Despite the increased focus on T1D antibody assays, data from methods comparison are limited. Moreover, the Islet Autoantibody Standardization Program (IASP) has recently observed discrepancies of positive/negative scores and the ranking of antibody levels across assays and formats across laboratories around the world, thus heightening the need for a more standardized procedure for these assays [18]. Hence, this study aimed to compare the diagnostic performance of the CLIA and ELISA in detecting the presence of three islet autoantibodies (GADA, ZnT8A, and IA-2A), with the purpose of identifying a more accurate, rapid, automated, and convenient method for the implementation of screening programs.

## 2. Materials and Methods

### 2.1. Assessment of Comparability Between Methods

A total of 104 serum specimens, covering the most clinically relevant range of each of Anti-GAD, Anti-ZnT8, and Anti-IA-2 antibody assays, were collected from a sample of children and adolescents with new-onset T1D or who were undergoing screening as first-degree relatives; these were collected in the PRECIMED-VR Biobank of the Regional Center for Pediatric Diabetes of Verona (Italy). Samples were collected from 182 patients (mean age: 8.9 years; 53% male). Detailed information regarding the antibody assay used for each patient is provided in Supplementary Table S1. Following blood collection, serum was separated within 2 h and aliquoted to prevent repeated freeze–thaw cycles. One aliquot was used for ELISA testing as part of the routine diagnostic workflow, while the second aliquot was stored at −80 °C for subsequent CLIA analysis. All analyses were conducted at the Laboratory Medicine Service of the University Hospital of Verona.

The study was cleared by the local Ethical Committee (Verona and Rovigo provinces; protocol number: 971CESC; date of approval: 25 July 2016). The analytical characteristics of the assays are summarized in Table 1.

The overall positive and negative percent agreement and Cohen’s κ coefficient were calculated to demonstrate the concordance between the two assays. Standard formulae have been used to calculate the percent agreement and relative sensitivity and specificity of the results from the CLIA method, considering ELISA as the reference standard. κ values lower than 0.40 mean poor agreement, values between 0.40 and 0.60 result in a moderate agreement, those between 0.60 and 0.80 depict a good agreement, and values over 0.80 demonstrate an excellent agreement. After the assessment of variable distribution using the Kolmogorov–Smirnov test, Spearman’s correlation analysis was used to identify the correlation between methods. Proportional and/or constant bias were assessed with Passing–Bablok regression and Bland–Altman analysis. Deviation from linearity was detected using the Cusum test. The statistical analysis was performed using SPSS 26.0 statistical software (IBM SPSS, Chicago, IL, USA) and Graph Pad 10.4.2 (San Diego, CA, USA).

### 2.2. Assessment of Precision and Linearity

Intra-assay variability was assessed by performing ten replicate measurements on aliquots of two serum pools for each class of major islet antibodies, with one pool having analyte concentrations below and the other above the established positivity cut-off. The resulting coefficients of variation (CVs) were compared with those provided by the CLIA manufacturer, which have been assessed with three human serum pools and internal control materials containing different analyte concentrations, each measured in duplicate. Linearity was evaluated by preparing serial dilutions (ranging from 1:10 to 10:1) of high-titer serum samples for GADA, ZnT8A, and IA-2A, each diluted with the corresponding low-titer serum pool specific to each class of major islet antibody. All dilutions were tested in triplicate, and values were averaged.

## 3. Results

### 3.1. Precision and Linearity

Table 2 shows the results of the imprecision study. The obtained values were generally consistent with those provided by the manufacturer, whose declared coefficients of variation (CVs) range from 0.5% to 4.5%.

Using the protocol described above, assay linearity was assessed within the range of 12 to 270 IU/mL for ZnT8A, 2.3 to 300 IU/mL for GADA, and 5 to 413 IU/mL for IA-2A. The assay demonstrated excellent linearity for ZnT8A and GADA (Spearman’s correlation coefficients of 0.992 and 0.999, respectively) and a good linearity for IA-2A (Spearman’s correlation coefficient of 0.962).

### 3.2. Comparability Between Methods

For each of the three autoantibodies tested (GADA, IA-2A, and ZnT8A), 104 serum samples were analyzed, spanning broad concentration ranges of each analyte (<1 to >2000 for ZnT8A; <0.2 to >2000 for GADA; and <2 to >1000 for IA-2A). The CLIA and ELISA methods showed strong cumulative agreement, with ZnT8A displaying the highest inter-assay concordance. The positive and negative agreement rates, as well as the Cohen’s kappa coefficient (based on dichotomizing the results at the manufacturers’ cut-off values), are summarized in Table 3.

**Figure 1:**
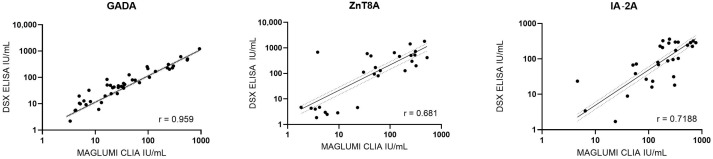
Spearman’s rank correlation between CLIA and ELISA measurements. CLIA: chemiluminescence immunoassay; ELISA: enzyme-linked immunosorbent assay; GADA: anti-glutamate decarboxylase autoantibody; IA-2A: anti-insulinoma antigen-2 autoantibody; ZnT8A: anti-zinc transporter protein 8 antibody. *n* = 52 for GADA, *n* = 30 for IA-2A, *n* = 31 for ZnT8A.

Passing–Bablok regression was used for samples within the measurable range of each assay in order to compare CLIA and ELISA measurements for the GADA, IA-2A, and ZnT8A antibodies. No significant deviation from linearity was observed in any of these comparisons (Cusum test; *p* > 0.05), indicating that a linear model was appropriate. The regression slopes revealed systematic differences between the methods. For GADA (*n* = 52) and ZnT8A (*n* = 31), the CLIA yielded systematically lower concentrations than the ELISA, with slopes significantly below 1 (95% CI: 0.6952–0.8242 and 0.4594–0.5427, respectively). Contrarily, CLIA values were higher than ELISA for IA-2A (*n* = 30), with a slope significantly above 1 (95% CI: 1.6891–2.6813). Spearman’s rank correlation analysis (Figure 1) yielded a strong correlation for GADA (r = 0.959) and moderately strong correlations for IA-2A (r = 0.719) and ZnT8A (r = 0.681). All correlation coefficients were statistically significant (*p* < 0.001).

**Figure 2:**
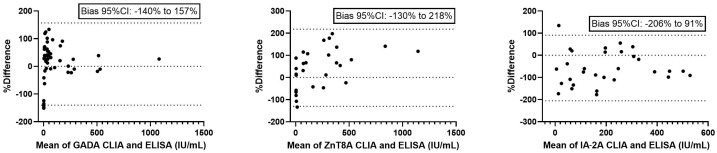
Bland–Altman agreement analysis between CLIA and ELISA measurements. CLIA: chemiluminescence immunoassay; ELISA: enzyme-linked immunosorbent assay; GADA: anti-glutamate decarboxylase autoantibody; IA-2A: anti-insulinoma antigen-2 autoantibody; ZnT8A: anti-zinc transporter protein 8 antibody. *n* = 52 for GADA, *n* = 30 for IA-2A, *n* = 31 for ZnT8A.

Bland–Altman analysis (Figure 2) was used to further assess the agreement between methods, revealing a relative dispersion of values, with broad 95% CIs.

## 4. Discussion

The screening for islet autoantibodies is now primarily conducted within programs targeting children, adolescents, and adults who are at an increased risk of developing T1D due to either having a first-degree relative with T1D or carrying a known high-risk HLA genotype [7,19]. The periodic monitoring of individuals who test positive for one or more islet autoantibodies is cost-effective and feasible using ELISA-based methods, given the relatively limited number of samples involved. Nevertheless, up to 90% of individuals who eventually develop T1D do not belong to these predefined at-risk groups; therefore, population-based screening initiatives are being launched, substantially increasing the number of samples to be analyzed. Regional screening programs could result in a shift from processing a few thousand samples per year to over twenty-fold samples annually. In this context, the use of traditional ELISA methods becomes impractical due to their limited scalability and lack of full automation. Hence, there is an urgent need for the availability of new, fully automated, and standardized platforms to efficiently monitor islet autoantibody status at the regional- and population-wide levels. Among the currently available alternative methods, the dissociation-enhanced lanthanide fluorescence immunoassay (DELFIA) and CLIA appear to be the most promising options [20,21].

In this study, we evaluated the analytical concordance and the quantitative agreement between the CLIA and ELISA for the measurement of anti-GAD, IA-2, and ZnT8 islet autoantibodies in a cohort of serum samples covering a broad dynamic range. Our findings offer valuable insights into the comparability of these two commonly used immunoassay platforms, highlighting both the strengths and potential limitations associated with their interchangeability. We also verified the precision and linearity of GADA, ZnT8A, and IA-2A CLIA assays on MAGLUMI 800.

The present study demonstrates a strong total agreement between CLIA and ELISA methods for the measurement of GADA, IA-2A, and ZnT8A autoantibodies, with Cohen’s kappa coefficients consistently indicating excellent concordance. Notably, ZnT8A exhibited the highest agreement between methods, while GADA showed a slightly lower concordance, particularly in terms of negative agreement. Despite these encouraging findings, method comparison analyses revealed significant proportional biases. Passing–Bablok regression proved that while a linear relationship was maintained, the CLIA systematically underestimated antibody concentrations for GADA and ZnT8A, and overestimated those for IA-2A compared to the ELISA. This trend was further supported by Bland–Altman analysis, which highlighted mean biases of +8% for GADA, +44% for ZnT8A, and −57% for IA-2A, indicating a substantial variation in both bias magnitude and direction across analytes. This suggests that although the CLIA and ELISA generate highly correlated data, the presence of systematic proportional differences precludes their direct interchangeability for quantitative assessment and longitudinal monitoring of patients’ data.

Our findings are in line with previous observations from Plebani et al. [22], who reported a detectable proportional bias between the SNIBE MAGLUMI™ 2000 Plus CLIA (Shenzhen, China) and the EUROIMMUN Anti-GAD ELISA (Padova, Italy) for GADA quantification. In their study, the CLIA method systematically underestimated GADA concentrations compared to ELISA, despite both assays being calibrated against the World Health Organization (WHO) 1st Reference Reagent 97/550. Similarly, in our analysis, Passing–Bablok regression and Bland–Altman plots revealed a significant proportional bias, with the CLIA underestimating GADA levels relative to the ELISA.

By extending the analysis performed by Plebani et al. [22] to ZnT8A and Ia-2A assays, we also showed a persistent substantial proportional bias between methods. In particular, the positive bias for ZnT8A and the negative bias for IA-2A highlight that the magnitude and direction of discrepancy may vary considerably depending on the specific islet autoantibodies analyzed.

This was the first study evaluating the comparability between the ELISA and CLIA for the three antibodies against three major pancreatic autoantigens. The major strengths of this study include the evaluation of a substantial number of samples across a wide dynamic range and the application of complementary statistical approaches (Passing–Bablok regression, Spearman’s correlation, and Bland–Altman analysis) to comprehensively assess method comparison.

However, of the 104 samples collected for each class of major islet antibodies, several results were below or above the measurable range of one or both of the ELISA and CLIA. Therefore, to avoid the misinterpretation of results, only those comprised within the measurable range of each assay were included in the quantitative analyses, which may have excluded extreme values that are potentially relevant in clinical practice.

Some limitations should be acknowledged in our investigation. First, this study assessed only three of the four autoantibodies recommended for autoimmune screening in type 1 diabetes. The evaluation of insulin autoantibodies (IAAs) was unfeasible due to the absence of positive samples among those analyzed during routine laboratory testing. The absence of clinical outcome data, such as disease stage at diagnosis, progression to clinical T1D, or impact on patient management, also limits the ability to draw definitive conclusions regarding the clinical significance of the observed analytical differences between the two methods. This is particularly relevant for values near diagnostic cut-offs or within the gray zone, where even minor deviations can influence classification outcomes. These considerations are especially critical in population-based screening contexts with low disease prevalence, where high specificity is essential to minimize false positive results. Although the ELISA was used as the reference method for comparison, it does not represent an established gold standard. Discrepancies between the ELISA and CLIA should not be interpreted as evidence of superiority of one method over the other, but rather as a reflection of their distinct analytical characteristics and limitations. The lack of a healthy control group assessed by both methods prevents a formal assessment of absolute specificity. Finally, the study was conducted at a single center, using specific commercial platforms, and did not include external validation, which limits the potential generalizability of the findings to other clinical settings and laboratory workflows.

## 5. Conclusions

In conclusion, our study demonstrates that the CLIA and ELISA for GADA, IA-2A, and ZnT8A exhibit strong agreement and high correlation coefficients. Although significant proportional biases between methods limit their quantitative interchangeability, our findings support the use of the CLIA in clinical practice for the detection of islet autoantibodies, including in the context of large-scale screening initiatives, due to their availability as a fully automated assay, rapidity, and overall reliability. Nonetheless, major efforts are still needed to increase the harmonization of serum islet autoantibody values generated using these two techniques.

## Figures and Tables

**Table 1:** Characteristics of the assays used in this investigation.

Test	ZnT8A		GADA		IA-2A	
	CLIA	ELISA	CLIA	ELISA	CLIA	ELISA
Reagent kit (manufacturer)	MAGLUMI Anti-ZnT8A (SNIBE, Shenzhen, China)	Elisa RSR ZnT8 Ab (RSR Limited, Cardiff, UK)	MAGLUMI Anti-GAD (SNIBE, Shenzhen, China)	Elisa RSR GAD Ab (RSR Limited, Cardiff, UK)	MAGLUMI Anti-IA-2 (SNIBE, Shenzhen, China)	Medizym anti-IA-2 M (MEDIPAN, Blankenfelde-Mahlow, Germany)
Instrument (manufacturer)	MAGLUMI 800 (SNIBE, Shenzhen, China)	DYNEX DSX (PANTEC, Torino, Italy)	MAGLUMI 800 (SNIBE, Shenzhen, China)	DYNEX DSX (PANTEC, Torino, Italy)	MAGLUMI 800 (SNIBE, Shenzhen, China)	DYNEX DSX (PANTEC, Torino, Italy)
Specimen storage stability	7 days (2–8 °C) 6 months (−20 °C)	Assay soon after sera separation or stored at or below −20 °C	14 days (2–8 °C) 3 months (−20 °C)	Assay soon after sera separation or stored at or below −20 °C	14 days (2–8 °C) 6 months (−20 °C)	3 days (2–8 °C) >3 days (−20 °C)
Min. required sample volume	20 μL	50 μL (25 μL × 2 wells)	50 μL	50 μL (25 μL × 2 wells)	100 μL	100 μL (50 μL × 2 wells)
Result interpretation	Negative: <10 AU/mL Positive: ≥10 AU/mL	Negative: <15 IU/mL Positive: ≥15 IU/mL	Negative: <10 IU/mL Positive: ≥10 IU/mL	Negative: <5 IU/mL Positive: ≥5 IU/mL	Negative: <10 IU/mL Positive: ≥10 IU/mL	Negative: <8 IU/mL Gray zone: 8–10 IU/mL Positive: >10 IU/mL
Analytical measurement range	1.00–2000 AU/mL	1.00–2000 IU/mL	0.2–2000 IU/mL	0.5–2000 IU/mL	2.00–1000 IU/mL	1–400 IU/mL

CLIA: chemiluminescence immunoassay; ELISA: enzyme-linked immunosorbent assay; GADA: anti-glutamate decarboxylase autoantibody; IA-2A: anti-insulinoma antigen-2 autoantibody; ZnT8A: anti-zinc transporter protein 8 antibody.

**Table 2:** Intra-assay imprecision of antibodies measured on serum pool samples below (low) and above (high) the positivity cut-off (10 replicate measurements).

MAGLUMI CLIA	Mean IU/L		CV%
GADA	Low	2.4	5.5
	High	177	2.3
IA-2A	Low	7.7	5.1
	High	125	0.5
ZnT8A	Low	8.03	2.4
	High	175	4.3

CLIA: chemiluminescence immunoassay; CV%: coefficient of variation; GADA: anti-glutamate decarboxylase autoantibody; IA-2A: anti-insulinoma antigen-2 autoantibody; ZnT8A: anti-zinc transporter protein 8 antibody.

**Table 3:** Agreement between the CLIA and ELISA.

	Overall Agreement	Positive Agreement	Negative Agreement	Cohen’s k Coefficient
IA-2A	96.2%	91.3%	100%	0.922
GADA	91.4%	100%	85%	0.827
ZnT8A	97.1%	100%	94.5%	0.943

CLIA: chemiluminescence immunoassay; GADA: anti-glutamate decarboxylase autoantibody; IA-2A: anti-insulinoma antigen-2 autoantibody; ZnT8A: anti-zinc transporter protein 8 antibody.

## Data Availability

Data will be available from the corresponding author upon reasonable request.

## Supplementary Materials

The following supporting information can be downloaded at: https://www.mdpi.com/article/10.3390/diagnostics15131695/s1, Table S1: Demographic characteristics of enrolled patients and type of testing conduced on each patient sample.

## Abbreviations

The following abbreviations are used in this manuscript:

T1D	type 1 diabetes
CD	celiac disease
DKA	diabetic ketoacidosis
ELISA	enzyme-linked immunosorbent assay
CLIA	chemiluminescence immunoassay
RIA	radioimmunoassay
GADA	anti-glutamate decarboxylase autoantibody
IA-2A	anti-insulinoma antigen-2 autoantibody
ZnT8A	anti-zinc transporter protein 8 antibody
IAA	anti-insulin autoantibody
IASP	Islet Autoantibody Standardization Program
DELFIA	dissociation-enhanced lanthanide fluorescence immunoassay
CVs	coefficients of variation
