# Supplementary material for: A Comparative Evaluation of the Chemiluminescence Immunoassay and ELISA for the Detection of Islet Autoantibodies in Type 1 Diabetes
Source: Diagnostics (Basel). 2025 Jul 3;15(13):1695. doi: 10.3390/diagnostics15131695 (PMC12249052; doi:10.3390/diagnostics15131695)
Supplement: Supplementary file 1 [file diagnostics-15-01695-s001.zip › diagnostics-3693460-supplementary.pdf]

Supplementary Table 1: Demographic characteristics of enrolled patients and type of testing conduced on each patient sample.

+: sample selected as positive for the presence of the corresponding antibody;

-: sample selected as negative for the presence of the corresponding antibody.

PATIENT ID	SEX	AGE	ZnT8 A	GAD A	IA-2 A
1	M	12	+	+	
2	F	12	+	+	
3	F	6	+	+	
4	F	12	+	+	
5	M	5	+	+	
6	M	14	+	+	
7	M	14	+	+	
8	M	17	+	+	
9	M	9	+	+	
10	M	14	+	+	
11	F	10	+	+	
12	F	6	+	+	
13	M	8			+
14	M	14			+
15	M	6	+		+
16	M	4	+		+
17	M	12	+		+
18	F	7	+		+
19	M	7		+	+
20	F	10		+	-
21	M	13		+	+
22	M	15		+	-
23	F	7		+	+
24	F	12		+	+
25	F	8		+	+
26	F	14		+	+
27	F	9			+
28	M	11	+	+	+
29	F	12	+	+	+
30	F	9	+	+	+
31	M	10	+	+	+
32	M	13	+	+	-
33	F	15	+	+	-
34	M	8	+	+	+
35	F	10	+	+	+
36	F	7	+	+	+
37	M	12	+	+	+

38	M	12	+	+	-
39	F	15	+	+	-
40	M	12	+	+	+
41	M	8	+	+	-
42	F	10	+	+	+
43	F	12	+	+	-
44	M	8	+		+
45	F	7	+		+
46	F	9	+		+
47	M	9			+
48	F	1			
49	M	1		+	
50	F	17			
51	M	5			
52	F	3			
53	M	6	+	+	
54	F	1	+	+	
55	M	6	+	+	
56	M	9	+	+	
57	M	13	+		
58	F	5	+		
59	F	6			+
60	M	1			+
61	F	4	+		+
62	M	3	+		+
63	F	6		+	-
64	F	3		+	-
65	M	1			+
66	M	10			+
67	F	2			
68	F	11			
69	M	7			
70	M	7	+	+	+
71	M	2	+	+	-
72	M	9	+	+	+
73	M	13	+	+	+
74	F	15	+	+	+
75	F	4	+	+	+
76	F	8	+	+	+
77	M	9	+	+	+
78	M	16	+	+	+
79	M	6			-
80	F	7			-

81	M	10			-
82	M	12			-
83	M	5			-
84	F	11			-
85	M	5			-
86	M	11			-
87	F	10			-
88	M	5			-
89	F	6			-
90	F	10			-
91	M	16			-
92	F	10			-
93	M	14			-
94	F	13			-
95	F	15			-
96	F	8			-
97	M	13			-
98	F	11			+
99	F	8			-
100	M	4			-
101	F	19			-
102	F	1			-
103	F	9			-
104	M	5			-
105	F	9			-
106	F	1			-
107	F	2			-
108	F	4			-
109	F	4			-
110	F	1			-
111	M	1			-
112	M	2			-
113	M	2			-
114	M	1			-
115	M	1			-
116	M	4			-
117	F	3			-
118	F	6			-
119	F	4			-
120	M	3			-
121	F	8			-
122	M	0			-
123	M	3			-

124	M	4			-
125	F	0			-
126	M	11			-
127	M	3			-
128	F	2			-
129	M	7			-
130	M	6			-
131	F	6	-	-	
132	M	15	-	-	
133	F	13	-	-	
134	M	16	-	-	
135	M	13	-	-	
136	M	14	-	-	
137	M	9	-	-	
138	F	16	-	-	
139	M	12	-	-	
140	F	9	-	-	
141	F	10	-	-	
142	F	7	-	-	
143	M	11	-	-	
144	F	17	-	-	
145	M	8	-	-	
146	F	14	-	-	
147	M	10	-	-	
148	M	18	-	-	
149	M	17	-	-	
150	F	11	-	-	
151	F	8	-	-	
152	F	12	-	-	
153	F	8	-	-	
154	M	10	-	-	
155	M	4	-	-	
156	F	12	-	-	
157	F	22	-	-	
158	M	14	-	-	
159	M	15	-	-	
160	F	10	-	-	
161	M	12	-	-	
162	M	17	-	-	
163	M	13	-	-	
164	M	12	-	-	
165	M	15	-	-	
166	F	7	-	-	

167	F	11	-	-	
168	F	11	-	-	
169	F	13	-	-	
170	M	9	-	-	
171	M	9	-	-	
172	M	11	-	-	
173	M	5	-	-	
174	F	13	-	-	
175	F	9	-	-	
176	M	3	-	-	
177	F	14	-	-	
178	M	4	-	-	
179	M	12	-	-	
180	M	16	-	-	
181	F	10	-	+	
182	F	15	-	-	
